# The learning curve in bladder MRI using VI-RADS assessment score during an interactive dedicated training program

**DOI:** 10.1007/s00330-022-08766-8

**Published:** 2022-04-02

**Authors:** Miguel Correia da Silva, Martina Pecoraro, Martina Lucia Pisciotti, Ailin Dehghanpour, Ali Forookhi, Sara Lucciola, Marco Bicchetti, Emanuele Messina, Carlo Catalano, Valeria Panebianco

**Affiliations:** 1 Department of Radiology, Centro Hospitalar Universitário de São João, Alameda Prof. Hernâni Monteiro, 4200-319 Porto, Portugal; 2 Department of Radiological Sciences, Oncology and Pathology, Sapienza University/Policlinico Umberto I, Viale del Policlinico 155, 00185 Rome, Italy; 3 Department of Radiological Sciences, Oncology and Pathology, Sapienza University/Policlinico Umberto I, Viale del Policlinico 155, 00161 Rome, Italy

**Keywords:** Internship and residency, Learning curve, Training program, Bladder cancer, Magnetic resonance imaging

## Abstract

**Objective:**

The purpose of the study was to evaluate the effect of an interactive training program on the learning curve of radiology residents for bladder MRI interpretation using the VI-RADS score.

**Methods:**

Three radiology residents with minimal experience in bladder MRI served as readers. They blindly evaluated 200 studies divided into 4 subsets of 50 cases over a 3-month period. After 2 months, the first subset was reassessed, resulting in a total of 250 evaluations. An interactive training program was provided and included educational lessons and case-based practice. The learning curve was constructed by plotting mean agreement as the ratio of correct evaluations per batch. Inter-reader agreement and diagnostic performance analysis were performed with kappa statistics and ROC analysis.

**Results:**

As for the VI-RADS scoring agreement, the kappa differences between pre-training and post-training evaluation of the same group of cases were 0.555 to 0.852 for reader 1, 0.522 to 0.695 for reader 2, and 0.481 to 0.794 for reader 3. Using VI-RADS ≥ 3 as cut-off for muscle invasion, sensitivity ranged from 84 to 89% and specificity from 91 to 94%, while the AUCs from 0.89 (95% CI:0.84, 0.94) to 0.90 (95% CI:0.86, 0.95). Mean evaluation time decreased from 5.21 ± 1.12 to 3.52 ± 0.69 min in subsets 1 and 5. Mean grade of confidence improved from 3.31 ± 0.93 to 4.21 ± 0.69, in subsets 1 and 5.

**Conclusion:**

An interactive dedicated education program on bladder MRI and the VI-RADS score led to a significant increase in readers’ diagnostic performance over time, with a general improvement observed after 100–150 cases.

**Key Points:**

*• After the first educational lesson and 100 cases were interpreted, the concordance on VI-RADS scoring between the residents and the experienced radiologist was significantly higher.*

*• An increase in the grade of confidence was experienced after 100 cases.*

*• We found a decrease in the evaluation time after 150 cases.*

**Supplementary Information:**

The online version contains supplementary material available at 10.1007/s00330-022-08766-8.

## Introduction

Bladder cancer (BCa) is the 10^th^ most common malignancy, with an approximately 550,000 new cases and 200,000 deaths worldwide [1], and has the highest lifetime economic burden per patient of all tumors, mainly due to hospital care-related costs [2].

In recent years, magnetic resonance imaging (MRI) has proven to be a reliable and accurate tool for BCa diagnosis and staging. In particular, the Vesical Imaging-Reporting and Data System (VI-RADS) score [3] was developed to provide a systematic and standardized approach in the acquisition, interpretation, and reporting of bladder MRI to differentiate muscle-invasive from non-muscle-invasive bladder cancer, aiming, among others, to reduce the heterogeneity in results between centers. Interestingly, beyond a high diagnostic performance, the VI-RADS assessment scoring showed a substantial inter-rater agreement among both experts and inexperienced radiologists [4–10]. As technology advances rapidly in radiology, medical schools and residency programs must adopt new methods of learning in order to implement substantial changes to the radiology curriculum delivery. Indeed, a strong emphasis should be placed upon reader education and experience in oncologic imaging. Up to now, numerous studies on MR imaging of the prostate showed variable diagnostic performance related to reader expertise [11]. Despite this, the optimal training curriculum for residents and fellows remains unclear. Learning curves for prostate MRI interpretation over time after dedicated reader training have not been extensively studied; nonetheless, an overall improvement in tumor detection accuracy was found after training [12–23]. To date, in contrast to prostate MRI, there have been no studies investigating the role of a learning program on the accuracy of bladder cancer staging using MRI and the VI-RADS score.

The objective of this study was to determine the learning curve of radiology residents in interpreting bladder MRI using the VI-RADS score during an interactive dedicated training program.

## Materials and methods

### Patient population and study design

**Fig. 1:**
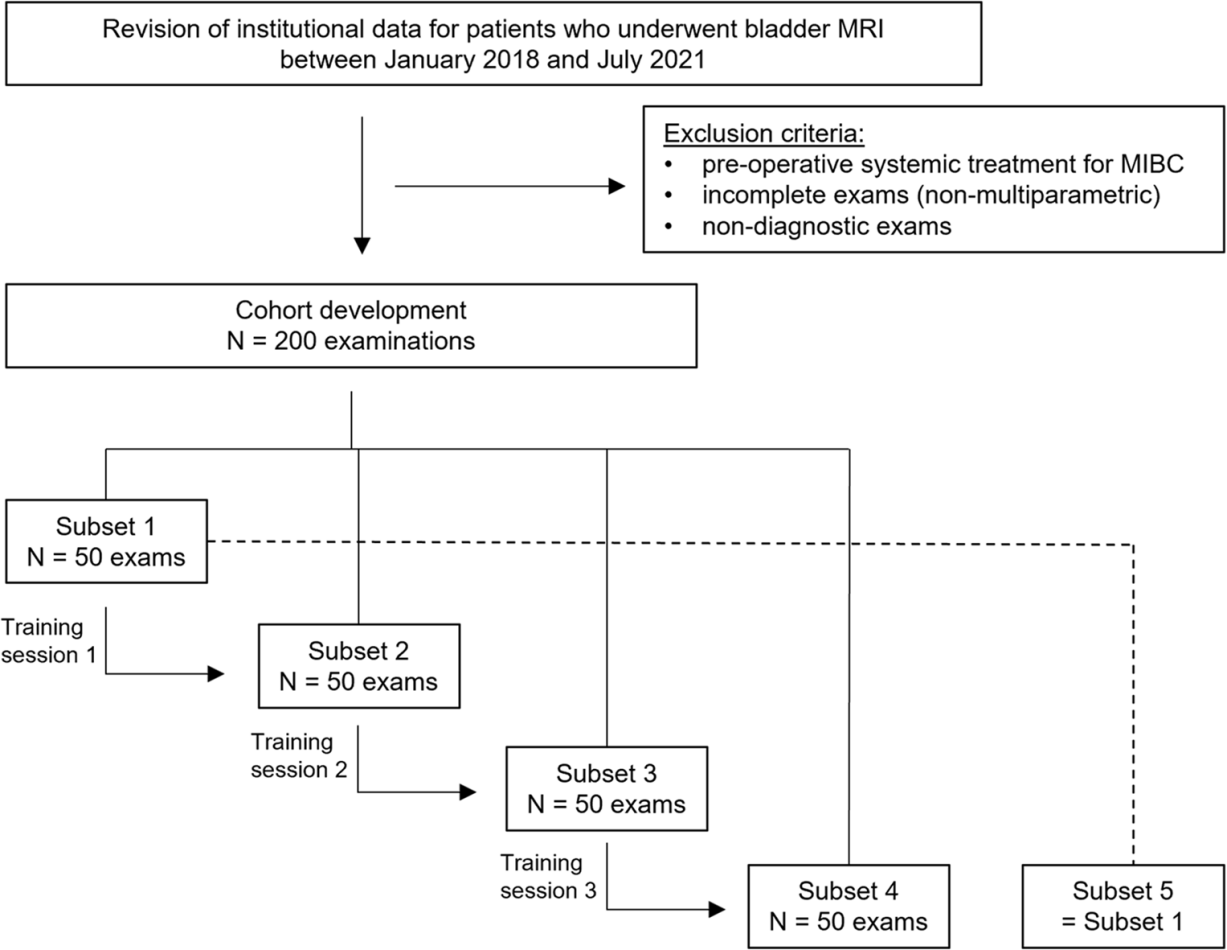
Study flow diagram. MIBC, muscle-invasive bladder cancer

This observational study was approved by our Institutional Review Board and the Ethical Committee. All patients were prospectively enrolled and were notified of the investigational nature of this study and gave their written informed consent. The study was conformed to the guidelines for good clinical practice in agreement with the ethical principles set forth in the latest version of the Declaration of Helsinki. Institutional data from 200 consecutive patients who underwent bladder MR imaging between January 2018 and July 2021 were analyzed. Patients who received pre-operative systemic treatment for muscle-invasive bladder cancer (MIBC) before imaging were excluded. Study design flowchart is presented in Fig. 1.

### MR imaging technique

All patients underwent the same MRI protocol using a 3-T scanner (General Electric Healthcare, Discovery 750); a small proportion of exams (3.5%) was acquired with a 1.5-T scanner (Siemens, Avanto) due to incompatibilities with medical devices (e.g., pacemakers) at 3-T magnetic field. The acquisition protocol, as per VI-RADS guidelines [3], included morphological multiplanar T2-weighted imaging (T2WI), on three planes (axial, coronal, and sagittal), according to lesion location; diffusion-weighted imaging (DWI) acquired in the axial plane with multiple *b* values (*b* = 100–800–2000) that were used to generate the apparent diffusion coefficient (ADC) map; dynamic contrast-enhanced (DCE) images were acquired in the axial plane with fat-suppression (3D T1 gradient echo), before and after gadolinium-based contrast media injection, with a temporal resolution of 8 s. An intramuscular antispasmodic agent was administered, if necessary, to reduce bladder wall motion artifacts, and patients were instructed to drink 500–1000 mL of water 30 min before the examination to obtain adequate bladder distension.

### Readers, imaging interpretation, and reference standard

Three radiology residents from two different institutions, at the first (M.L.P., A.D.) and fourth year (M.C.S.) of training, with a general understanding of body MRI but minimal experience in bladder MRI interpretation (less than 20 cases in total), served as readers. They independently evaluated 200 consecutive studies in four equal subsets (batches) of 50 cases over a 3-month period. After a 2-month memory extinction period, the first subset was re-evaluated, resulting in a total of 250 assessments. All examinations were anonymized, and the readers were blinded to clinical information including patients presenting signs and symptoms, previous imaging studies, history of cystoscopy, and tumor biopsy.

Image analysis was performed using the VI-RADS assessment score, by evaluating first the morphological T2W images, then the DWI/ADC map sequence, and finally the DCE images. Whenever more than one lesion was identified, the overall VI-RADS score provided corresponded to either the lesion with the highest VI-RADS category or, in cases where the scores were equal, the lesion with the largest size. Additionally, the evaluation time, the grade of confidence, and the image quality were recorded. Evaluation time corresponded to the period spent in interpreting the exam and the thinking process to formulate the score rounded to the nearest minute. Grade of confidence was documented according to a 5-point Likert scale (Supplementary Table 1). Also, readers were asked to rate image quality on a 3-point scale according to T2WI, DWI, and DCE diagnostic quality standards based on an institutional-specific algorithm (Supplementary Table 2). Interpretation by an expert urogenital radiologist with 10 years of bladder MRI experience (V.P.) and a cumulative reading experience of 500 bladder MRIs using VI-RADS score, was considered the reference standard (RS).

### Educational interventions in the dedicated training program

The dedicated training program had a duration of 3 months. Prior to the reading of the first subset of cases, the readers were given an overview lecture on bladder MRI, followed by a review and interpretation of five cases from the Picture Archive and Communication System (PACS). Additional studying material was provided to the readers and included recent and insightful articles on these topics. During the period between batches 1 and 2, a more experienced resident provided an educational lecture on the VI-RADS assessment score, with a focus on the assessment of each scoring category. An interactive session involving revision of cases, that the readers found challenging in the first groups and/or had discordant scoring, was provided between batches 2 and 3 by a resident with four years of experience in urogenital MRI. Finally, a bladder MRI expert provided an advanced bladder cancer imaging presentation between the last two batches, with radiologic and pathologic correlation. Questions were encouraged during every lesson to aid readers’ learning. After recording the case scoring for each batch, the readers were shown the reference standard reports, in order to review the cases incorrectly evaluated and further improve their learning process.

### Statistical analysis

Descriptive statistics were used to summarize overall, per-batch and per-reader VI-RADS score category assignment, grade of confidence (GC), evaluation time (ET) and image quality (IQ) using number and percentages (VI-RADS score), and the mean and standard deviation (GC, ET, and IQ). By plotting mean agreement as a ratio of correct evaluations per batch, a learning curve was constructed for the three readers. Inter-reader agreement analysis was performed with Cohen’s kappa statistics, for each VI-RADS score by each of the three readers pairs. The diagnostic performance of each reader was assessed by means of receiver operating characteristic curve analysis. The overall and per-batch AUC values were obtained based upon single VI-RADS scoring and a conversion of VI-RADS to above or below a cut-off of 3 (cases were split into < 3 vs ≥ 3) for the evaluation of muscle-invasiveness. Overall and per-batch sensitivity and specificity were calculated to assess the performance of each reader per batch. *p* < 0.05 was considered to indicate a significant difference for all hypothesis tests. Analyses were performed using SPSS, version 27 (IBM).

## Results

The population cohort included 200 patients of which 73.5% were male and 26.5% were female (M:F ratio = 3:1) and the median age was 70 years (IQR 62–77). Out of the 200 exams, 13 (6.5%) had no identifiable lesion. Of the remaining 187 (93.5%) that were assigned a VI-RADS score by the expert radiologist and considered the reference standard, 13 (6.5%) were VI-RADS 1, 101 (50.5%) were VI-RADS 2, 13 (6.5%) were VI-RADS 3, 25 (12.5%) were VI-RADS 4, and 35 (17.5%) were VI-RADS 5. Overall VI-RADS score distribution by reader and per evaluated subset is summarized in Table 1.

**Table 1:** VI-RADS score distribution by rater for each subset. *VI-RADS*, Vesical Imaging-Reporting and Data System

	Subset 1	Subset 2	Subset 3	Subset 4	Subset 5	Total
	*N* = 50	*N* = 50	*N* = 50	*N* = 50	*N* = 50	*N* = 250
**Reference standard**						
No lesion	1 (2.0%)	3 (6.0%)	6 (12.0%)	3 (6.0%)	1 (2.0%)	14 (5.6%)
VI-RADS 1	4 (8.0%)	3 (6.0%)	4 (8.0%)	2 (4.0%)	4 (8.0%)	17 (6.8%)
VI-RADS 2	25 (50.0%)	27 (54.0%)	20 (40.0%)	29 (58.0%)	25 (50.0%)	126 (50.4%)
VI-RADS 3	2 (4.0%)	5 (10.0%)	2 (4.0%)	4 (8.0%)	2 (4.0%)	15 (6.0%)
VI-RADS 4	9 (18.0%)	4 (8.0%)	8 (16.0%)	4 (8.0%)	9 (18.0%)	34 (13.6%)
VI-RADS 5	9 (18.0%)	8 (16.0%)	10 (20.0%)	8 (16.0%)	9 (18.0%)	44 (17.6%)
**Reader 1**						
No lesion	4 (8.0%)	4 (8.0%)	8 (16.0%)	3 (6.0%)	3 (6.0%)	22 (8.8%)
VI-RADS 1	4 (8.0%)	3 (6.0%)	6 (12.0%)	2 (4.0%)	3 (6.0%)	18 (7.2%)
VI-RADS 2	20 (40.0%)	29 (58.0%)	17 (34.0%)	30 (60.0%)	26 (52.0%)	122 (48.8%)
VI-RADS 3	2 (4.0%)	3 (6.0%)	3 (6.0%)	6 (12.0%)	2 (4.0%)	16 (6.4%)
VI-RADS 4	13 (26.0%)	1 (2.0%)	8 (16.0%)	3 (6.0%)	7 (14.0%)	32 (12.8%)
VI-RADS 5	7 (14.0%)	10 (20.0%)	8 (16.0%)	6 (12.0%)	9 (18.0%)	40 (16.0%)
**Reader 2**						
No lesion	1 (2.0%)	4 (8.0%)	8 (16.0%)	4 (8.0%)	1 (2.0%)	18 (7.2%)
VI-RADS 1	5 (10.0%)	4 (8.0%)	5 (10.0%)	2 (4.0%)	3 (6.0%)	19 (7.6%)
VI-RADS 2	23 (46.0%)	28 (56.0%)	16 (32.0%)	28 (56.0%)	27 (54.0%)	122 (48.8%)
VI-RADS 3	9 (18.0%)	2 (4.0%)	6 (12.0%)	3 (6.0%)	3 (6.0%)	23 (9.2%)
VI-RADS 4	4 (8.0%)	3 (6.0%)	5 (10.0%)	2 (4.0%)	9 (18.0%)	23 (9.2%)
VI-RADS 5	8 (16.0%)	9 (18.0%)	10 (20.0%)	11 (22.0%)	7 (14.0%)	45 (18.0%)
**Reader 3**						
No lesion	5 (10.0%)	3 (6.0%)	8 (16.0%)	3 (6.0%)	1 (2.0%)	20 (8.0%)
VI-RADS 1	1 (2.0%)	4 (8.0%)	4 (8.0%)	2 (4.0%)	3 (6.0%)	14 (5.6%)
VI-RADS 2	20 (40.0%)	25 (50.0%)	18 (36.0%)	30 (60.0%)	26 (52.0%)	119 (47.6%)
VI-RADS 3	8 (16.0%)	6 (12.0%)	4 (8.0%)	3 (6.0%)	6 (12.0%)	27 (10.8%)
VI-RADS 4	4 (8.0%)	3 (6.0%)	6 (12.0%)	3 (6.0%)	6 (12.0%)	22 (8.8%)
VI-RADS 5	12 (24.0%)	9 (18.0%)	10 (20.0%)	9 (18.0%)	8 (16.0%)	48 (19.2%)

### Learning curve

**Fig. 2:**
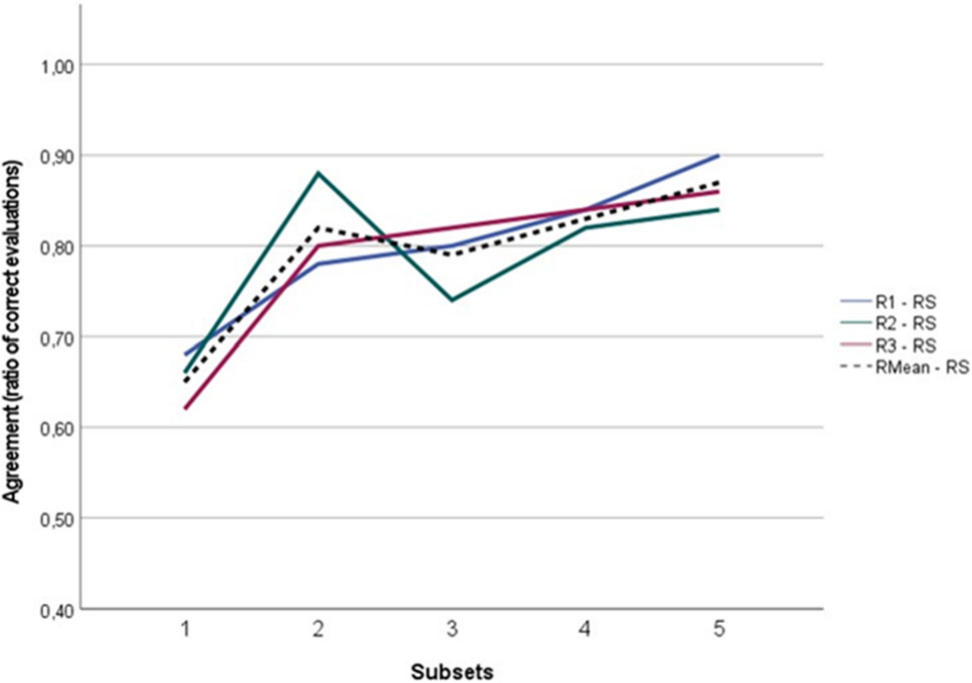
Reader’s learning curve for VI-RADS scoring over time, based on the ratio of concordant evaluations between readers and reference standard, per subset of images. R1, reader 1; R2, reader 2; R3, reader 3; RS, reference standard; RMean, reader’s mean

The learning curve over time on VI-RADS scoring, for the three readers, is presented in Fig. 2. The mean ratio of concordant VI-RADS scoring between readers and the reference standard improved steeply from 65% in batch 1 to 82% in batch 2. In subsequent batches, the case evaluation showed slight improvement, up to 87% seen in batch 5. The same increasing trend was noted regarding the kappa coefficient for the VI-RADS agreement between readers and the reference standard (Table 2). As such, for subsets 1 and 4 respectively, the *k* was 0.555 and 0.739 for reader 1, 0.522 and 0.712 for reader 2, and 0.481 and 0.737 for reader 3. The *k* coefficient differences between pre-training evaluation (subset 1) and post-training evaluation of the same group of patients (subset 5) were respectively 0.555 to 0.852 for reader 1, 0.522 to 0.695 for reader 2, and 0.481 to 0.794 for reader 3.

**Table 2:** Summary statistics for reader agreement**.** *RS*, reference standard

	Cohen’s kappa (k)					
	Subset 1	Subset 2	Subset 3	Subset 4	Subset 5	Overall
Reader 1 - RS	0.555*	0.656*	0.742*	0.739*	0.852*	0.712*
Reader 2 - RS	0.522*	0.815*	0.668*	0.712*	0.759*	0.695*
Reader 3 - RS	0.481*	0.704*	0.766*	0.737*	0.794*	0.697*
Mean	0.519	0.725	0.725	0.729	0.801	0.701

*p < 0.001

### Diagnostic accuracy

**Fig. 3:**
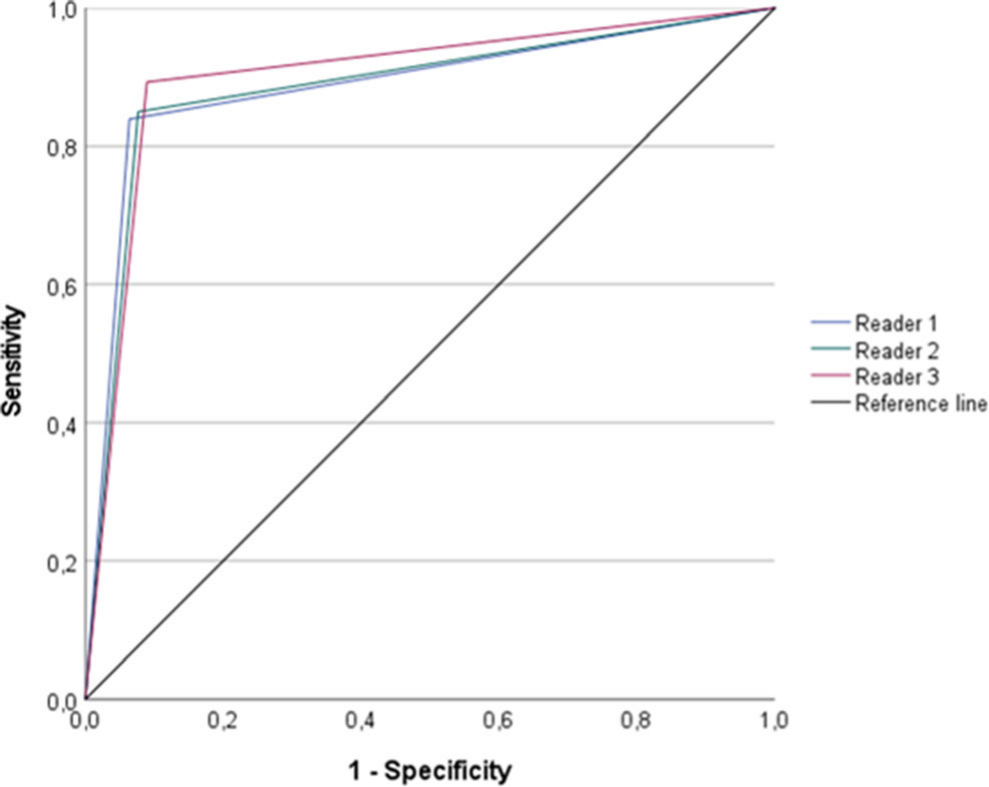
ROC curve demonstrating the AUCs for the three readers in detecting bladder cancer muscle invasiveness. ROC, reader characteristics curve; AUC, area under the curve

The performance of each reader in scoring single VI-RADS assessment categories, for the detection of bladder cancer muscle invasiveness (using a VI-RADS ≥ 3 as cut-off), and for the identification of absence of lesions was measured based on overall evaluations (Table 3) and on a per-batch basis (Supplementary Tables 3–9). Using VI-RADS ≥ 3 as cut-off, the sensitivity ranged from 84 to 89% and the specificity from 91 to 94%, across the three readers. The AUCs ranged from 0.89 (95% CI: 0.84, 0.94) to 0.90 (95% CI: 0.86, 0.95). Fig. 3 shows the receiver operating characteristic curves for the three readers on the detection of muscle-invasive bladder cancer.

**Table 3:** Overall diagnostic performance for each reader on single VI-RADS scoring, muscle invasiveness detection, and absence of lesion identification. *VI-RADS*, Vesical Imaging-Reporting and Data System; *AUC*, area under the curve; *CI*, confidence interval; *Sens*, sensitivity; *Spec*, specificity; *R1*, reader 1; *R2*, reader 2; *R3*, reader 3

Overall	AUC (95% CI)	Sens (%)	Spec (%)
no lesion	R1 – 0.98 (0.97–1.00)	100	97
	R2 – 0.99 (0.98–1.00)	100	98
	R3 – 0.95 (0.87–1.00)	93	97
VI-RADS 1	R1 – 0.94 (0.85–1.00)	88	99
	R2 – 0.93 (0.84–1.00)	88	98
	R3 – 0.85 (0.72–0.98)	70	100
VI-RADS 2	R1 – 0.91 (0.87–0.95)	90	83
	R2 – 0.88 (0.84–0.93)	87	81
	R3 – 0.89 (0.85–0.94)	87	82
VI-RADS 3	R1 – 0.71 (0.55–0.88)	47	96
	R2 – 0.59 (0.43–0.76)	27	92
	R3 – 0.76 (0.61–0.91)	60	92
VI-RADS 4	R1 – 0.75 (0.64–0.86)	56	94
	R2 – 0.70 (0.60–0.81)	44	96
	R3 – 0.67 (0.56–0.78)	38	96
VI-RADS 5	R1 – 0.84 (0.76–0.93)	73	96
	R2 – 0.93 (0.87–0.99)	89	97
	R3 – 0.95 (0.90–0.99)	93	97
Muscle invasiveness	R1 – 0.89 (0.84–0.94)	84	94
	R2 – 0.89 (0.84–0.94)	85	92
	R3 – 0.90 (0.86–0.95)	89	91

### Evaluation time

**Fig. 4:**
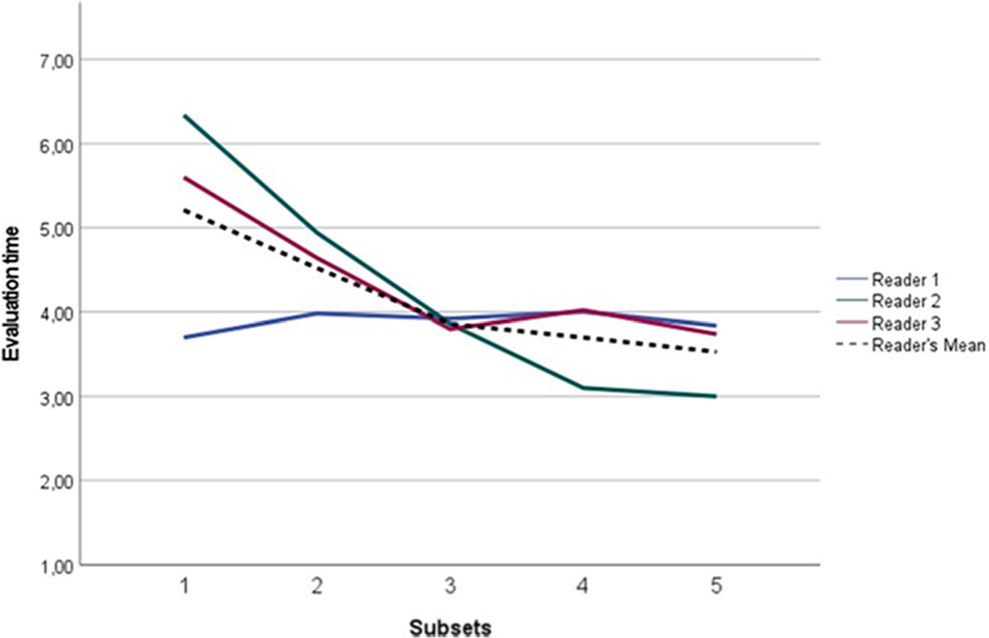
Reader’s mean evaluation time for each subset (in minutes). R1, reader 1; R2, reader 2; R3, reader 3; RS, reference standard; RMean, reader’s mean

The mean reader evaluation time decreased as subsequent batches were assessed from 5.21 ± 1.12 min in subset 1 to 3.52 ± 0.69 min in subset 5 (Table 4 and Fig. 4). A statistically significant reduction was found in mean evaluation time between subsets 1 and 4/5, between subsets 1 and 2, and between subsets 2 and 3 (*p* < 0.001), not between subsets 3 and 4 (*p* = 0.32), nor 4 and 5 (*p* = 0.45). For reader 1, no significant change in evaluation time of any consecutive subsets was shown (*p* ≥ 0.106). For reader 2, a statistically significant decrease between subsets 1 and 2 and subsets 2 and 3 (*p* < 0.001), as well as between subsets 3 and 4 (*p* = 0.03) was found, while no difference was noted between subsets 4 and 5 (*p* = 0.656). For reader 3, a statistically significant reduction between subsets 1 and 2 and subsets 2 and 3 (*p* < 0.001) was found, despite having no differences between subsets 3 and 4 (*p* = 0.258), nor 4 and 5 (*p* = 0.93).

**Table 4:** Reader mean + SD evaluation time for each subset recorded in minutes. *SD*, standard deviation

	Subset 1	Subset 2	Subset 3	Subset 4	Subset 5
Reader 1	3.70 ± 0.79	3.98 ± 0.92	3.92 ± 0.92	3.98 ± 0.92	3.84 ± 0.87
Reader 2	6.34 ± 2.19	4.94 ± 1.70	3.86 ± 1.23	3.10 ± 1.28	3.00 ± 0.93
Reader 3	5.60 ± 1.46	4.64 ± 1.03	3.80 ± 1.01	4.02 ± 0.92	3.74 ± 0.72
All readers	5.21 ± 1.12	4.52 ± 0.91	3.86 ± 0.82	3.70 ± 0.77	3.52 ± 0.69

### Grade of confidence

**Fig. 5:**
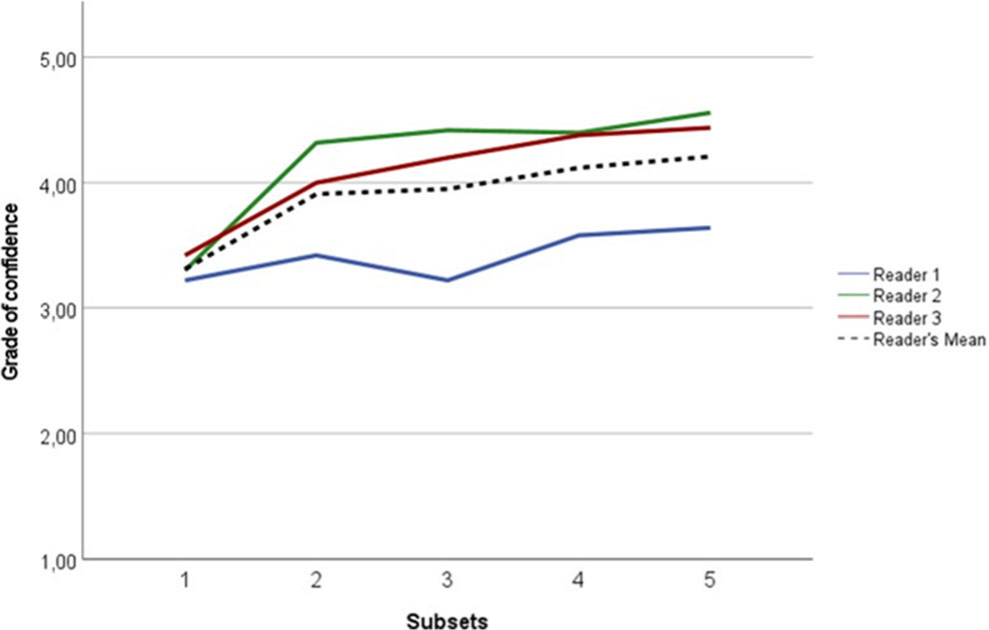
Reader’s mean grade of confidence score for each subset (5-point assessment scale). R1, reader 1; R2, reader 2; R3, reader 3; RS, reference standard; RMean, reader’s mean.

Mean grade of confidence improved as subsequent batches were assessed from 3.31 ± 0.93 in subset 1 to 4.21 ± 0.69 in subset 5 (Table 5 and Fig. 5). A statistically significant increase was found in mean grade of confidence between subsets 1 and 4/5 and subsets 1 and 2 (*p* < 0.001). Mean grade of confidence was not different between the remaining consecutive subsets (*p* ≥ 0.294). For reader 1, no significant difference was found between any subsets (*p* ≥ 0.58). For readers 2 and 3, a significant increase in grade of confidence was found between subsets 1 and 2 (*p* < 0.001; *p* = 0,044; respectively), with no differences between other consecutive subsets (*p* ≥ 0.216; *p* ≥ 0,438; respectively).

**Table 5:** Reader mean + SD grade of confidence score (5-point scale) for each subset. *SD*, standard deviation

	Subset 1	Subset 2	Subset 3	Subset 4	Subset 5
Reader 1	3.22 ± 1.23	3.42 ± 1.11	3.22 ± 1.11	3.58 ± 0.95	3.64 ± 0.94
Reader 2	3.30 ± 1.09	4.32 ± 0.71	4.42 ± 0.88	4.40 ± 0.73	4.56 ± 0.54
Reader 3	3.42 ± 1.55	4.00 ± 1.28	4.20 ± 1.29	4.38 ± 1.05	4.44 ± 1.16
All readers	3.31 ± 0.93	3.91 ± 0.77	3.95 ± 0.90	4.12 ± 0.72	4.21 ± 0.69

### Image quality

**Fig. 6:**
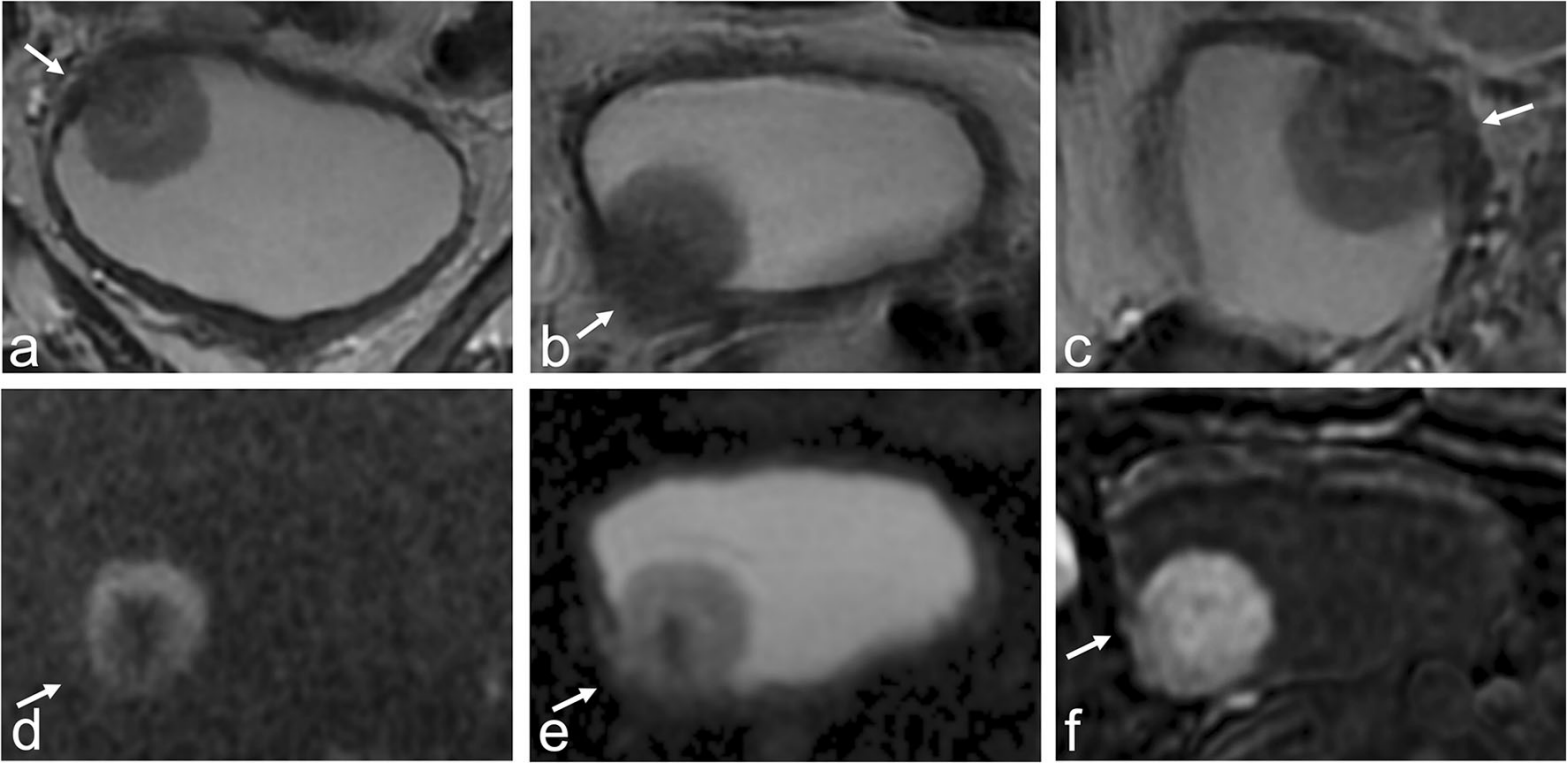
Case example of a 67-year-old male. **a** Coronal T2WI showing a pedunculated bladder tumor at the bladder dome extending to the right lateral wall with a clearly uninterrupted muscularis propria layer (arrow); **b** axial T2WI showing an apparently interrupted muscularis propria (arrow); **c** sagittal T2WI showing an equivocal alteration of the muscularis propria (arrow); **d**, **e** DWI and ADC map showing no interruption of the muscularis propria layer (arrows), and the “inchworm sign”, which is usually indicative, as in this case, of NMIBC; **f** DCE MRI showing the integrity of the *muscularis propria* layer and the inner layer sign (arrow), both indicative of NMIBC. The images were incorrectly scored as a VI-RADS 3 and 4 by the inexperienced readers during the first interpretation batch, probably due to the non-optimal quality acquisition; however, MRI was correctly scored with an overall VI-RADS 2 in batch 5, given the higher reader experience. T stage after TURBT identified HG-T1 urothelial carcinoma. T2WI, T2-weighted imaging; VI-RADS, Vesical Imaging-Reporting and Data System; DWI, diffusion-weighted imaging; ADC, apparent diffusion coefficient; DCE, dynamic contrast-enhanced; NMIBC, non-muscle-invasive bladder cancer; TURBT, trans-urethral resection of bladder tumor; HG, high-grade

When the image quality was minimal (IQ1), the overall VI-RADS score agreement between readers and the reference standard was moderate (*k* = 0.503 for reader 1; *k* = 0.508 for reader 2; *k* = 0.603 for reader 3; *p* < 0.001). When the image quality was scored as optimal (IQ3), the overall VI-RADS score agreement was substantial, with (*k* = 0.739 for reader 1; *k* = 0.726 for reader 2; *k* = 0.713 for reader 3; *p* < 0.001). The results on *k* statistics and image quality are shown in Table 6. Summary on overall quality assessment scoring is provided in Supplementary Table 10. Figure 6 illustrates an example of case that was incorrectly classified during batch 1 and correctly scored during batch 5.

**Table 6:** VI-RADS agreement between readers and the reference standard based on image quality (3-point scale). *RS*, reference standard

	Cohen’s kappa	
	Image quality 1	Image quality 3
Reader 1 - RS	0.503*	0.739*
Reader 2 - RS	0.508*	0.726*
Reader 3 - RS	0.630*	0.713*
Mean	0.547	0.726

*p < 0.001

## Discussion

Different studies have shown that readers’ education and training are key factors in oncologic imaging and for training future radiologists [11]. Despite this, no evidence exists on the effect of an interactive learning program on the reader performance in bladder MRI and the VI-RADS score. The purpose of this study was to assess the learning curve and to determine how three radiology residents performed when interpreting bladder MRI using VI-RADS assessment scoring as part of a dedicated interactive training program, in which 200 cases of bladder MRI were divided into four sets. In between the subsets, frontal lessons, dedicated case-review, and tutoring sessions were provided, followed by a final re-assessment of the first subset of cases (batch 5).

We observed a significant increase in concordance between the VI-RADS scoring of the residents, compared to the experienced radiologist, after the first two batches of training (100 cases) showing a steep improvement from 65 to 82% followed by a plateau; reader 2 experience a drop in improvement in batch 3, probably due to the high number of cases in agreement with the RS in batch 2; by revising batch 2 cases for reader 2 we noticed that the number of cases scored as low quality was very low (*n* = 5), which might explain such high agreement ratio. The steep improvement in the bladder MRI outcomes was likely linked to the educational intervention that focused on providing general information on VI-RADS assessment scoring.

In the agreement analysis, this trend was also observed when looking at *k* coefficient that improved from a mean of 0.519 in batch 1 to 0.801 in batch 5. These results might suggest that providing an overview on the VI-RADS criteria combined with a sample number of cases (100–150) might lead to acceptable results, highlighting the strength of this reporting and data system for a standardized approach to bladder MRI interpretation. This is in line with the ESUR/ESUI consensus statement establishing 100 supervised cases as the minimum number of prostate MRI before independent reporting can be performed for clinically significant prostate cancer detection [24].

Despite the slower and lower trends in subsequent subsets, the learning curve of the residents continued to rise, illustrating the need for more advanced and prolonged training. Differing from our experience, Rosenkrantz et al did not report a significant improvement in interpreting prostate MRI using PI-RADS v. 2.0 in the group of readers receiving continual feedback [12]. A more recent study found that online courses significantly improved the sensitivity in detecting prostate cancer on MRI using PI-RADS score [25].

As for overall bladder MRI and VI-RADS scoring diagnostic performance, we found promising results in the assessment of the likelihood of muscle invasion (VI-RADS 1-2 vs 3-4-5). Indeed, using VI-RADS ≥ 3 as cut-off, across the three readers, the overall sensitivity ranged from 84 to 89% and overall specificity from 91 to 94%. The overall AUCs ranged from 0.89 (95% CI: 0.84, 0.94) to 0.90 (95% CI: 0.86, 0.95) going from the lowest AUC of 0.82 (95% CI: 0.69–0.95) in batch 1 to 0.96 (95% CI: 0.89–1.00) in batch 5. In similar reports, two groups demonstrated a higher AUC (from 0.52 to 0.66; *p* < 0.001) in detecting prostate cancer after an interactive training course [15] and higher detection rate of the index prostate cancer (from 74.2 to 87.7%; *p* = 0.003) [14].

To what regards evaluation time, a particularly relevant topic for today’s heavily loaded radiology departments, the two residents at the first year of training demonstrated a significant decrease in mean interpretation time after the first 150 cases (mean overall ET: 5.21 min in subset 1 to 3.52 min in subset 5; *p <* 0.001). A similar outcome was observed in another study where authors found that the mean reader evaluation time decreased significantly from 95.2–99.0 s in subsets 1–2 to 66.1–65.8 s in subsets 3–4 (*p* < 0.001), when readers received feedback [12]. However in our study, as previously mentioned, this trend was only observed for the first-year residents, indicating that general exposure to MR imaging may lead to shorter assessment periods, regardless of previous exposure to bladder MRI. As such, the fourth-year resident did not show differing mean timeframes during the training program, which suggests no association between specific bladder MRI training and reduced evaluation time.

Considering the grade of confidence of the readers, our results led to the same conclusions of Garcia-Reyes et al who found significant improvements between pre- and post-education evaluations of prostate MRIs (3.75 to 4.22 on a scale from 1 to 5) [14]. In our study, mean overall confidence ranged from 3.31 in subset 1 to 4.21 in subset 5 (5-point scale). Confidence in reporting, specifically in assessing the likelihood of tumor invasion of the muscularis propria, is of utmost importance as it can dramatically change and guide patients’ management. We point out that throughout the study, for both the per-subset and the overall results, the percentage of VI-RADS score 3 (equivocal cases) was lower than or equal to 11%, which may differentiate this system from other -RADS [26] in which the number of assigned indeterminate cases is higher, having a strong clinical impact in bladder cancer imaging.

Even though it is not directly related to the effects of a training program, image quality clearly influences the diagnostic performance of radiologists. This was confirmed in our study in which we found that the agreement between the residents and the experienced radiologist was higher when the images were perceived as high-quality exams. This might have influenced the outcome of the learning process as the mean overall k coefficient increased from 0.547 (low IQ) to 0.726 (high IQ). We hypothesize that expertise might be proven advantageous particularly in poor quality exams, but this thesis warrants further research.

These findings have several implications regarding trainee education in bladder MRI interpretation. This study demonstrated the risk of interpreting bladder MRIs without prior experience or training, which is why we do not recommend reading exams without basic knowledge of MRIs and specifically of bladder MRI. Efforts should be made to guarantee that every radiologist reading bladder MRI has an acceptable number of interpreted exams (100 cases according to our results) along with a proper understanding of the VI-RADS criteria. Radiology curricula could be improved by including training on bladder MRI, aiding to a standardized management using the VI-RADS criteria, and leading to a more value-based service to patients with bladder cancer.

The following limitations are acknowledged: first, we did not investigate the pathology corroboration of the results; second, some heterogeneity was observed in-between batches as we included subsequent patients and did not evenly distribute a homogenous number of each VI-RADS score throughout the groups of patients; third, the MR images were acquired with a highly performing 3 Tesla MRI scan, in a tertiary referral center, which might negatively impact the reproducibility of our findings; fourth, our three subjects received training, and no control group was formed, which might be a source of bias. Finally, the applicability of the proposed training program in trainee daily routine is partly currently limited due to the COVID-19 pandemics. However, most of the activities included in the program could be easily organized on an online learning platform.

In conclusion, an interactive dedicated reader education program on bladder MRI and the VI-RADS score was associated with a significant increase in readers’ diagnostic performance over time. A general improvement was observed after 100–150 cases, which might be proposed as a cut-off to reach learning programs. These findings may represent a useful experience to improve and shape future fellowship programs and radiology curricula.

## Supplementary information

ESM 1 (DOCX 38 kb)

## Abbreviations

ADC: Apparent diffusion coefficient
BCa: Bladder cancer
DCE: Dynamic contrast-enhanced
DWI: Diffusion-weighted imaging
ESUR/ESUI: European Society of Urogenital Radiology / EAU Section of Urologic Imaging
ET: Evaluation time
GC: Grade of confidence
IQ: Image quality
MIBC: Muscle-invasive bladder cancer
MRI: Magnetic resonance imaging
NMIBC: Non-muscle-invasive bladder cancer
PACS: Picture Archiving and Communication System
PI-RADS: Prostate Imaging-Reporting and Data System
RS: Reference Standard
SD: Standard deviation
T2WI: T2-weighted imaging
VI-RADS: Vesical Imaging-Reporting and Data System

## Declarations

### Guarantor

The scientific guarantor of this publication is Valeria Panebianco.

### Statistics and biometry

No complex statistical methods were necessary for this paper.

### Methodology

• prospective

• observational

• performed at one institution
