# Supplementary material for: The learning curve in bladder MRI using VI-RADS assessment score during an interactive dedicated training program
Source: Eur Radiol. 2022 Apr 2;32(11):7494–503. doi: 10.1007/s00330-022-08766-8 (PMC8976109; doi:10.1007/s00330-022-08766-8)
Supplement: Supplementary file 1 — (DOCX 38 kb) [file 330_2022_8766_MOESM1_ESM.docx]

**ELECTRONIC SUPPLEMENTARY MATERIAL (ESM)**

**Supplementary Tables**

| **Grade of confidence score** | **Criteria** |
| --- | --- |
| 1 | < 20% confidence on interpretation |
| 2 | 20 - 40% confidence on interpretation |
| 3 | 40 - 60% confidence on interpretation |
| 4 | 60 - 80% confidence on interpretation |
| 5 | > 80% confidence on interpretation |

**Supplementary Table 1** – Grade of confidence scoring criteria.

| **Image quality score** | **Criteria** |
| --- | --- |
| 1 | T2WI, DWI and DCE are below the minimum standards for diagnostic quality |
| 2 | T2WI and/or DWI and/or DCE are of acceptable diagnostic quality |
| 3 | T2WI, DWI and DCE are of optimal diagnostic quality |

**Supplementary Table 2** – Image quality scoring criteria. T2WI, T2-weighted imaging; DWI, diffusion-weighted imaging; DCE, dynamic contrast-enhanced.

| nO LESION | AUC (95% ci) | sens (%) | spec  (%) |
| --- | --- | --- | --- |
| bATCH 1 | R1 0.97 (0.91-1.00) | 100 | 94 |
|  | r2 1.00 (1.00-1.00) | 100 | 100 |
|  | R3 0.96 (0.89-1.00) | 100 | 92 |
| bATCH 2 | R1 0.99 (0.96-1.00) | 100 | 98 |
|  | r2 0.99 (0.96-1.00) | 100 | 98 |
|  | R3 0.82 (0.49-1.00) | 67 | 98 |
| bATCH 3 | R1 0.98 (0.94-1.00) | 100 | 95 |
|  | r2 0.98 (0.94-1.00) | 100 | 95 |
|  | R3 0.98 (0.94-1.00) | 100 | 95 |
| BATCH 4 | R1 1.00 (1.00-1.00) | 100 | 100 |
|  | r2 0.99 (0.96-1.00) | 100 | 98 |
|  | R3 1.00 (1.00-1.00) | 100 | 100 |
| BATCH 5 | R1 0.98 (0.93-1.00) | 100 | 96 |
|  | r2 1.00 (1.00-1.00) | 100 | 100 |
|  | R3 1.00 (1.00-1.00) | 100 | 100 |
| OVERALL | R1 0.98 (0.97-1.00) | 100 | 97 |
|  | r2 0.99 (0.98-1.00) | 100 | 98 |
|  | R3 0.95 (0.87-1.00) | 93 | 97 |

**Supplementary Table 3.** Diagnostic performance for each reader on assessing no lesion per batch. AUC, area under the curve; CI, confidence interval; Sens, sensitivity; Spec, specificity; R1, reader 1; R2, reader 2; R3, reader 3.

| VI-RADS 1 | AUC (95% ci) | sens (%) | spec  (%) |
| --- | --- | --- | --- |
| bATCH 1 | R1 0.86 (0.61-1.00) | 75 | 98 |
|  | R2 0.85 (0.60-1.00) | 75 | 96 |
|  | R3 0.63 (0.30-0.96) | 25 | 100 |
| bATCH 2 | R1 1.00 (1.00-1.00) | 100 | 100 |
|  | R2 0.99 (0.96-1.00) | 100 | 98 |
|  | R3 0.99 (0.96-1.00) | 100 | 98 |
| bATCH 3 | R1 0.98 (0.94-1.00) | 100 | 96 |
|  | r2 0.99 (0.96-1.00) | 100 | 98 |
|  | R3 1.00 (1.00-1.00) | 100 | 100 |
| BATCH 4 | R1 1.00 (1.00-1.00) | 100 | 100 |
|  | R2 1.00 (1.00-1.00) | 100 | 100 |
|  | R3 0.74 (0.29-1.00) | 50 | 98 |
| BATCH 5 | R1 0.88 (0.62-1.00) | 65 | 100 |
|  | R2 0.88 (0.62-1.00) | 65 | 100 |
|  | R3 0.88 (0.62-1.00) | 65 | 100 |
| OVERALL | R1 0.94 (0.85-1.00) | 88 | 99 |
|  | R2 0.93 (0.84-1.00) | 88 | 98 |
|  | R3 0.85 (0.72-0.98) | 70 | 100 |

**Supplementary Table 4.** Diagnostic performance for each reader on assessing VI-RADS score 1 per batch. AUC, area under the curve; CI, confidence interval; Sens, sensitivity; Spec, specificity; R1, reader 1; R2, reader 2; R3, reader 3.

| VI-RADS 2 | AUC (95% ci) | sens (%) | spec  (%) |
| --- | --- | --- | --- |
| bATCH 1 | R1 0.86 (0.75-0.97) | 76 | 96 |
|  | R2 0.80 (0.67-0.93) | 76 | 98 |
|  | R3 0.82 (0.70-0.94) | 72 | 92 |
| bATCH 2 | R1 0.88 (0.77-0.98) | 93 | 83 |
|  | R2 0.94 (0.86-1.00) | 96 | 91 |
|  | R3 0.88 (0.78-0.99) | 85 | 91 |
| bATCH 3 | R1 0.93 (0.83-1.00) | 85 | 100 |
|  | R2 0.90 (0.79-1.00) | 80 | 100 |
|  | R3 0.95 (0.87-1.00) | 90 | 100 |
| BATCH 4 | R1 0.89 (0.79-1.00) | 93 | 86 |
|  | R2 0.86 (0.75-0.97) | 86 | 86 |
|  | R3 0.85 (0.74-0.97) | 90 | 81 |
| BATCH 5 | R1 0.98 (0.94-1.00) | 100 | 95 |
|  | R2 0.92 (0.83-1.00) | 100 | 88 |
|  | R3 0.94 (0.86-1.00) | 100 | 92 |
| OVERALL | R1 0.91 (0.87-0.95) | 90 | 83 |
|  | R2 0.88 (0.84-0.93) | 87 | 81 |
|  | R3 0.89 (0.85-0.94) | 87 | 82 |

**Supplementary Table 5.** Diagnostic performance for each reader on assessing VI-RADS score 2 per batch. AUC, area under the curve; CI, confidence interval; Sens, sensitivity; Spec, specificity; R1, reader 1; R2, reader 2; R3, reader 3.

| VI-RADS 3 | AUC (95% ci) | sens (%) | spec  (%) |
| --- | --- | --- | --- |
| bATCH 1 | R1 0.74 (0.29-1.00) | 50 | 98 |
|  | R2 0.67 (0.24-1.00) | 50 | 98 |
|  | R3 0.94 (0.86-1.00) | 100 | 87 |
| bATCH 2 | R1 0.58 (0.29-0.87) | 20 | 96 |
|  | R2 0.59 (0.30-0.88) | 20 | 98 |
|  | R3 0.77 (0.50-1.00) | 60 | 93 |
| bATCH 3 | R1 0.63 (0.28-1.00) | 50 | 96 |
|  | R2 0.44 (0.72-0.80) | 100 | 88 |
|  | R3 0.46 (0.77-0.84) | 100 | 92 |
| BATCH 4 | R1 0.71 (0.39-1.00) | 50 | 91 |
|  | R2 0.60 (0.28-0.93) | 25 | 96 |
|  | R3 0.74 (0.42-1.00) | 50 | 98 |
| BATCH 5 | R1 1.00 (1.00-1.00) | 100 | 100 |
|  | R2 0.73 (0.28-1.00) | 50 | 96 |
|  | R3 0.96 (0.90-1.00) | 100 | 92 |
| OVERALL | R1 0.71 (0.55-0.88) | 47 | 96 |
|  | R2 0.59 (0.43-0.76) | 27 | 92 |
|  | R3 0.76 (0.61-0.91) | 60 | 92 |

**Supplementary Table 6.** Diagnostic performance for each reader on assessing VI-RADS score 3 per batch. AUC, area under the curve; CI, confidence interval; Sens, sensitivity; Spec, specificity; R1, reader 1; R2, reader 2; R3, reader 3.

| VI-RADS 4 | AUC (95% ci) | sens (%) | spec  (%) |
| --- | --- | --- | --- |
| bATCH 1 | R1 0.75 (0.55-0.94) | 67 | 88 |
|  | R2 0.59 (0.36-0.81) | 22 | 95 |
|  | R3 0.52 (0.31-0.73) | 11 | 93 |
| bATCH 2 | R1 0.49 (0.20-0.78) | 100 | 98 |
|  | R2 0.88 (0.62-1.00) | 75 | 100 |
|  | R3 0.74 (0.42-1.00) | 50 | 98 |
| bATCH 3 | R1 0.78 (0.57-0.99) | 63 | 93 |
|  | R2 0.59 (0.36-0.82) | 25 | 93 |
|  | R3 0.65 (0.42-0.89) | 38 | 93 |
| BATCH 4 | R1 0.74 (0.42-1.00) | 50 | 98 |
|  | R2 0.75 (0.42-1.00) | 50 | 100 |
|  | R3 0.74 (0.42-1.00) | 50 | 98 |
| BATCH 5 | R1 0.82 (0.63-1.00) | 67 | 98 |
|  | R2 0.80 (0.60-0.99) | 67 | 93 |
|  | R3 0.77 (0.56-0.99) | 56 | 98 |
| OVERALL | R1 0.84 (0.76-0.93) | 73 | 96 |
|  | R2 0.93 (0.87-0.99) | 89 | 97 |
|  | R3 0.95 (0.90-0.99) | 93 | 97 |

**Supplementary Table 7.** Diagnostic performance for each reader on assessing VI-RADS score 4 per batch. AUC, area under the curve; CI, confidence interval; Sens, sensitivity; Spec, specificity; R1, reader 1; R2, reader 2; R3, reader 3.

| VI-RADS 5 | AUC (95% ci) | sens (%) | spec  (%) |
| --- | --- | --- | --- |
| bATCH 1 | R1 0.69 (0.47-0.91) | 44 | 93 |
|  | R2 0.88 (0.71-1.00) | 78 | 98 |
|  | R3 0.90 (0.77-1.00) | 89 | 90 |
| bATCH 2 | R1 0.90 (0.76-1.00) | 88 | 93 |
|  | R2 0.99 (0.96-1.00) | 100 | 98 |
|  | R3 0.91 (0.77-1.00) | 88 | 95 |
| bATCH 3 | R1 0.84 (0.66-1.00) | 70 | 93 |
|  | R2 0.94 (0.83-1.00) | 90 | 97 |
|  | R3 1.00 (1.00-1.00) | 100 | 100 |
| BATCH 4 | R1 0.88 (0.69-1.00) | 75 | 100 |
|  | R2 0.96 (0.92-1.00) | 100 | 93 |
|  | R3 0.99 (0.96-1.00) | 100 | 98 |
| BATCH 5 | R1 0.93 (0.80-1.00) | 89 | 98 |
|  | R2 0.89 (0.72-1.00) | 78 | 100 |
|  | R3 0.94 (0.82-1.00) | 89 | 100 |
| OVERALL | R1 0.84 (0.76-0.93) | 73 | 96 |
|  | R2 0.93 (0.87-0.99) | 89 | 97 |
|  | R3 0.95 (0.90-0.99) | 93 | 97 |

**Supplementary Table 8.** Diagnostic performance for each reader on assessing VI-RADS score 5 per batch. AUC, area under the curve; CI, confidence interval; Sens, sensitivity; Spec, specificity; R1, reader 1; R2, reader 2; R3, reader 3.

| MI | AUC (95% ci) | sens (%) | spec  (%) |
| --- | --- | --- | --- |
| bATCH 1 | R1 0.88 (0.78-0.99) | 90 | 87 |
|  | R2 0.82 (0.69-0.95) | 80 | 83 |
|  | R3 0.85 (0.74-0.97) | 90 | 80 |
| bATCH 2 | R1 0.82 (0.68-0.96) | 71 | 94 |
|  | R2 0.91 (0.80-1.00) | 82 | 100 |
|  | R3 0.90 (0.79-1.00) | 88 | 91 |
| bATCH 3 | R1 0.89 (0.79-1.00) | 85 | 93 |
|  | R2 0.90 (0.80-1.00) | 90 | 90 |
|  | R3 0.92 (0.82-1.00) | 90 | 93 |
| BATCH 4 | R1 0.88 (0.75-1.00) | 81 | 94 |
|  | R2 0.86 (0.74-0.99) | 81 | 91 |
|  | R3 0.88 (0.75-1.00) | 81 | 94 |
| BATCH 5 | R1 0.95 (0.87-1.00) | 90 | 100 |
|  | R2 0.93 (0.85-1.00) | 90 | 97 |
|  | R3 0.96 (0.89-1.00) | 95 | 97 |
| OVERALL | R1 0.89 (0.84-0.94) | 84 | 94 |
|  | R2 0.89 (0.84-0.94) | 85 | 92 |
|  | R3 0.90 (0.86-0.95) | 89 | 91 |

**Supplementary Table 9.** Diagnostic performance for each reader on assessing muscle-invasiveness (VI-RADS score ≥ 3) per batch. AUC, area under the curve; CI, confidence interval; MI, muscle invasiveness; Sens, sensitivity; Spec, specificity; R1, reader 1; R2, reader 2; R3, reader 3.

|  | **Subset 1** | **Subset 2** | **Subset 3** | **Subset 4** | **Subset 5** | **Total** |
| --- | --- | --- | --- | --- | --- | --- |
|  | **N = 50** | **N = 50** | **N = 50** | **N = 50** | **N = 50** | **N = 250** |
| **Reader 1** |  |  |  |  |  |  |
|  |  |  |  |  |  |  |
| Image Quality 1 | 10 (20.0%) | 13 (26.0%) | 14 (28.0%) | 8 (16.0%) | 7 (14.0%) | 52 (20.8%) |
| Image Quality 2 | 20 (40.0%) | 21 (42.0%) | 24 (48.0%) | 23 (46.0%) | 23 (46.0%) | 111 (44.4%) |
| Image Quality 3 | 20 (40.0%) | 16 (32.0%) | 12 (24.0%) | 19 (38.0%) | 20 (40.0%) | 87 (34.8%) |
|  |  |  |  |  |  |  |
| **Reader 2** |  |  |  |  |  |  |
|  |  |  |  |  |  |  |
| Image Quality 1 | 4 (8.0%) | 5 (10.0%) | 8 (16.0%) | 3 (6.0%) | 4 (8.0%) | 24 (9.6%) |
| Image Quality 2 | 26 (52.0%) | 35 (70.0%) | 23 (46.0%) | 29 (58.0%) | 26 (52.0%) | 139 (55.6%) |
| Image Quality 3 | 20 (40.0%) | 10 (20.0%) | 19 (38.0%) | 18 (36.0%) | 20 (40.0%) | 87 (34.8%) |
|  |  |  |  |  |  |  |
| **Reader 3** |  |  |  |  |  |  |
|  |  |  |  |  |  |  |
| Image Quality 1 | 13 (26.0%) | 1 (2.0%) | 1 (2.0%) | 2 (4.0%) | 13 (26.0%) | 30 (12.0%) |
| Image Quality 2 | 11 (22.0%) | 18 (36.0%) | 23 (46.0%) | 27 (54.0%) | 12 (24.0%) | 91 (36.4%) |
| Image Quality 3 | 26 (52.0%) | 31 (62.0%) | 26 (52.0%) | 21 (42.0%) | 25 (50.0%) | 129 (51.6%) |

**Supplementary Table 10.** Image quality score distribution by rater for each subset.
